# The complete mitochondrial genomes of three parasitic nematodes of birds: a unique gene order and insights into nematode phylogeny

**DOI:** 10.1186/1471-2164-14-414

**Published:** 2013-06-21

**Authors:** Guo-Hua Liu, Renfu Shao, Jia-Yuan Li, Dong-Hui Zhou, Hu Li, Xing-Quan Zhu

**Affiliations:** 1 State Key Laboratory of Veterinary Etiological Biology, Key Laboratory of Veterinary Parasitology of Gansu Province, Lanzhou Veterinary Research Institute, Chinese Academy of Agricultural Sciences, Lanzhou, Gansu Province, 730046, People’s Republic of China; 2 College of Veterinary Medicine, Hunan Agricultural University, Changsha, Hunan Province, 410128, People’s Republic of China; 3 Genecology Research Centre, University of the Sunshine Coast, Queensland, 4558, Australia; 4 Department of Entomology, China Agricultural University, Beijing, 100193, People’s Republic of China

**Keywords:** Mitochondrial genome, Ascaridia, Nematode, Gene arrangement, Phylogeny

## Abstract

**Background:**

Analyses of mitochondrial (mt) genome sequences in recent years challenge the current working hypothesis of Nematoda phylogeny proposed from morphology, ecology and nuclear small subunit rRNA gene sequences, and raise the need to sequence additional mt genomes for a broad range of nematode lineages.

**Results:**

We sequenced the complete mt genomes of three *Ascaridia* species (family Ascaridiidae) that infest chickens, pigeons and parrots, respectively. These three *Ascaridia* species have an identical arrangement of mt genes to each other but differ substantially from other nematodes. Phylogenetic analyses of the mt genome sequences of the *Ascaridia* species, together with 62 other nematode species, support the monophylies of seven high-level taxa of the phylum Nematoda: 1) the subclass Dorylaimia; 2) the orders Rhabditida, Trichinellida and Mermithida; 3) the suborder Rhabditina; and 4) the infraorders Spiruromorpha and Oxyuridomorpha. Analyses of mt genome sequences, however, reject the monophylies of the suborders Spirurina and Tylenchina, and the infraorders Rhabditomorpha, Panagrolaimomorpha and Tylenchomorpha. Monophyly of the infraorder Ascaridomorpha varies depending on the methods of phylogenetic analysis. The Ascaridomorpha was more closely related to the infraorders Rhabditomorpha and Diplogasteromorpha (suborder Rhabditina) than they were to the other two infraorders of the Spirurina: Oxyuridorpha and Spiruromorpha. The closer relationship among Ascaridomorpha, Rhabditomorpha and Diplogasteromorpha was also supported by a shared common pattern of mitochondrial gene arrangement.

**Conclusions:**

Analyses of mitochondrial genome sequences and gene arrangement has provided novel insights into the phylogenetic relationships among several major lineages of nematodes. Many lineages of nematodes, however, are underrepresented or not represented in these analyses. Expanding taxon sampling is necessary for future phylogenetic studies of nematodes with mt genome sequences.

## Background

Nematodes (also called roundworms) are an extremely diverse group of bilateral animals with an estimate of 1–10 million species although only ~25,000 species have been described [1]. Nematode taxonomy has traditionally been formed from morphology and ecology, e.g. the early systems proposed by Schneider (1866) [2] on somatic musculature and by Cobb (1919) [3] on stoma armature, and the system proposed by Filipjev (1929) [4] on the presence and absence of zooparasitism. Chitwood (1937) [5] proposed initially the bipartite phylogenetic and taxonomic system based on the presence and absence of phasmids; this system was further elaborated later by Maggenti (1963, 1983) [6,7] based on pharyngeal structure and excretory systems. However, due to the lack of homologous characters and informative fossil records, and the extensive convergent evolution, it has been extremely difficult to derive a consistent phylogenetic and taxonomic framework for the phylum Nematoda from morphological and ecological characters [8-12]. In the past two decades, nematode phylogeny and taxonomy have been revised with analyses of the nuclear small subunit (SSU) rRNA gene sequences [12-14]. The current working hypothesis of Nematoda phylogeny incorporated evidence from morphology, ecology and SSU rRNA gene sequence analyses [13,14].

The mitochondrial (mt) genomes of bilateral animals usually contain 37 genes and a control region on a circular chromosome, ~16 kb in size [15-17]. Sequences of individual mt genes and whole mt genomes are widely used to infer phylogenetic relationships among animals at different taxonomic levels [18,19]. In a number of cases, arrangement of mt genes has also been used to resolve long-standing phylogenetic relationships that could not be resolved by other means [20-22].

Recently, mt genome sequences have also been analyzed to understand the phylogenetic relationships among nematodes. Kang *et al*. [23] inferred the Nematoda phylogeny with the mt genome sequences of 25 species; Park *et al*. [24] and Sultana *et al*. [25] expanded further the analysis to include 36 and 41 species respectively. A major difference between mt genome phylogenies and the current working hypothesis of the Nematoda is on the relationship among four infraorders: Ascaridomorpha, Rhabditomorpha, Spiruromorpha and Oxyuridomorpha. In the current working hypothesis, Ascaridomorpha, Spiruromorpha and Oxyuridomorpha are in the suborder Spirurina whereas Rhabditomorpha is in the suborder Rhabditina [13,14]. Furthermore, Ascaridomorpha is most closely related to Spiruromorpha, whereas Oxyuridomorpha is sister to Ascaridomorpha + Spiruromorpha + Rhigonematomorph. In the phylogenies inferred from mt genome sequences, however, Ascaridomorpha is most closely related to Rhabditomorpha; in most analyses, Oxyuridomorpha is sister to Ascaridomorpha + Rhabditomorpha, whereas Spiruromorpha is sister to the group that contains Ascaridomorpha, Rhabditomorpha and Oxyuridomorpha [23,24]. Due to limited taxon sampling, however, the novel phylogenetic relationships inferred from mt genome sequences in these studies need to be interpreted with caution and further test with more taxa from a wide range of lineages is necessary.

In this study, we sequenced the complete mt genomes of three *Ascaridia* species in the family Ascaridiidae of the infraorder Ascaridomorpha. *Ascaridia* species are among the most prevalent and pathogenic parasitic nematodes found in domestic and wild birds and have a worldwide distribution [26]. These *Ascaridia* species share an identical arrangement of mt genes to each other but differ substantially from those of other nematodes. We inferred the phylogenetic relationships with the complete mt genome sequences of the *Ascaridia* species and 62 other nematode species that have been sequenced to date. Our analyses support the close relationship between Ascaridomorpha and Rhabditomorpha, and provide novel insights into other phylogenetic relationships in the Nematoda.

## Results

### Mitochondrial genomes of three *Ascaridia* species

**Figure 1:**
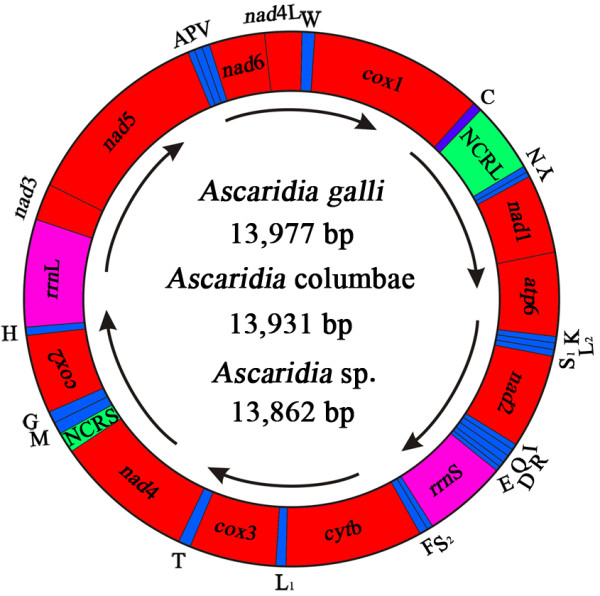
**The mitochondrial genomes of three *Ascaridia *species*.*** All genes are on the same DNA strand and are transcribed clockwise. Protein-coding and rRNA genes are indicated with the standard nomenclature. tRNA genes are indicated with the one-letter code of their corresponding amino acids. There are two tRNA genes for leucine: L_1_ for codons CUN and L_2_ for UUR; and two tRNA genes for serine: S_1_ for codons AGN and S_2_ for UCN. “NCRL” refers to the large non-coding region. “NCRS” refers to a small non-coding region.

The complete mt genomes of *A. galli*, *A. columbae* and *Ascaridia* sp. (GHL-2012) were 13,977 bp, 13,931 bp and 13,862 bp long, respectively (GenBank accession numbers JX624728, JX624729 and JX624730). Each mt genome contains 12 protein-coding genes, 22 tRNA genes and two rRNA genes on a circular chromosome (Figure 1). As in most other nematodes, *atp8* gene is not present in the mt genome of *A. galli*, *A. columbae* and *Ascaridia* sp. (GHL-2012); all mt genes are transcribed from the same direction. TTT is used as the initiation codon in *nad6* gene in the mt genome of *A. galli*. TTT was previously reported to be the initiation codon for *cox2, cytb, nad4, nad3, nad2* and *cox3* genes in the mt genome of *Strongyloides stercoralis* (Strongyloididae), but not for any other Ascaridida nematodes. All of the 12 protein-coding genes have complete termination codons, TAA or TAG. The tRNA genes in mt genomes of *A. galli*, *A. columbae* and *Ascaridia* sp. (GHL-2012) range from 51 to 76 bp. The tRNA-Ser^(AGN)^ and tRNA-Ser^(UCN)^ have truncated secondary structures; these two tRNAs lack a DHU stem but possess a TΨC loop. In all other 20 tRNA genes, the TΨC arm and variable loop are replaced with a TV replacement loop. There are two non-coding regions (NCR) in the mt genomes of *A. galli*, *A. columbae* and *Ascaridia* sp. (GHL-2012) (Table 1). The longer NCR (NCRL) is between *trnC* gene and *trnN* gene, and is 610 bp (*A. galli*), 563 bp (*A. columbae*) and 566 bp [*Ascaridia* sp. (GHL-2012)] respectively. The shorter NCR (NCRS) is between *nad4* and *trnM* gene, and is 157 bp (*A. galli*), 91 bp (*A. columbae*) and 101 bp [*Ascaridia* sp. (GHL-2012)] respectively. A NCR at this location, i.e. between *nad4* and *trnM*, is also present in the mt genomes of most other nematodes [27,28].

**Table 1:** **List of annotated mitochondrial genes and regions of *Ascaridia galli*, *Ascaridia columbae *and *Ascaridia *sp**

**Gene/region**	**Position/length (bp)**			**Start/stop codon**			**Anticodons**
	***A. galli***	***A. columbae***	***Ascaridia *sp.**	***A. galli***	***A.columbae***	***Ascaridia *sp.**	
*cox1*	1-1563 (1563)	1-1563 (1563)	1-1581 (1581)	ATG/TAA	ATG/TAA	GTG/TAG	
tRNA-Cys (C)	1565-1620 (56)	1565-1620 (56)	1562-1616 (55)				GAT
Non-coding region (NCL)	1621-2230 (610)	1623-2183 (563)	1617-2182 (566)				
tRNA-Asn (N)	2231 -2284 (54)	2184-2241 (58)	2183-2239 (57)				GTT
tRNA-Tyr (Y)	2286-2344 (59)	2246-2303 (58)	2240-2295 (56)				GTA
*nad1*	2342-3217 (876)	2301-3176 (876)	2298-3167 (870)	TTG/TAA	TTG/TAA	GTT/TAG	
*atp6*	3217-3813 (597)	3176-3772 (597)	3170-3766 (597)	ATA/TAA	ATA/TAA	TTG/TAG	
tRNA-Lys (K)	3816-3876 (61)	3790-3853 (64)	3772-3833 (62)				TTT
tRNA-Leu ^UUR^ (L_2_)	3876-3931(56)	3853-3907 (55)	3831-3886 (56)				TAA
tRNA-Ser ^AGN^ (S_1_)	3932-3982 (51)	3908-3962 (55)	3887-3939 (53)				GCT
*nad2*	3986-4828 (843)	3966-4808 (843)	3943-4779 (837)	TTG/TAA	TTG/TAA	TTG/TAG	
tRNA-Ile (I)	4832-4891 (60)	4823-4884 (62)	4781-4841 (61)				GAT
tRNA-Arg (R)	4896-4950 (55)	4888-4944 (57)	4845-4900 (56)				ACG
tRNA-Gln (Q)	4951-5005 (55)	4945-4998 (54)	4901-4954 (54)				TTG
tRNA-Asp (D)	5005-5063 (59)	5011-5070 (60)	4971-5025 (55)				GTC
tRNA-Glu (E)	5065-5125 (61)	5069-5126 (58)	5025-5083 (59)				TTC
*rrn*S	5123-5823 (701)	5124-5826 (703)	5084-5774 (691)				
tRNA-Ser ^UCN^ (S_2_)	5826-5880 (55)	5837-5891 (55)	5781-5835 (55)				TGA
tRNA-Phe (F)	5883-5939 (57)	5898-5953 (56)	5841-5897 (57)				GAA
*cytb*	5964-7067 (1104)	5975-7075 (1101)	5919-7019 (1101)	ATG/TAA	GTT/TAA	GTT/TAG	
tRNA-Leu ^CUN^ (L_1_)	7067-7122 (56)	7077-7134 (58)	7020-7075 (56)				TAG
*cox3*	7144-7914 (771)	7154-7918 (765)	7097-7864 (768)	TTG/TAG	TTG/TAA	TTG/TAA	
tRNA-Thr (T)	7889-7944 (56)	7899-7956 (58)	7845-7900 (56)				TAG
*nad4*	7939-9174 (1236)	7951-9186 (1236)	7901-9130 (1230)	GTG/TAG	ATA/TAG	TTG/TAG	
Non-coding region(NCR)	9175-9331 (157)	9187-9277 (91)	9131-9231 (101)				
tRNA-Met (M)	9345-9407 (63)	9288-9352 (65)	9232-9294 (63)				CAT
tRNA-Gly (G)	9407-9462 (56)	9355-9410 (56)	9296-9351 (56)				TCC
*cox2*	9463-10158 (696)	9411-10106 (696)	9364-10050 (687)	TTG/TAG	TTG/TAG	ATG/TAG	
tRNA-His (H)	10163-10218 (56)	10113-10167 (55)	10049-10105 (57)				GTG
*rrn*L	10211-11166 (956)	10167-11121 (955)	10103-11055 (953)				
*nad3*	11167-11502 (336)	11119-11454 (336)	11053-11391 (339)	TTG/TAA	ATA/TAA	TTG/TAA	
*nad5*	11502-13082 (1581)	11451-13031 (1581)	11394-12968 (1575)	ATA/TAA	ATA/TAG	ATT/TAG	
tRNA-Ala (A)	13085-13139 (55)	13031-13085 (55)	12968-13022 (55)				TGC
tRNA-Pro (P)	13140-13197 (58)	13094-13149 (56)	13026-13082 (57)				TGG
tRNA-Val (V)	13197-13252 (56)	13149-13205 (57)	13082-13137 (56)				TAC
*nad6*	13253-13687 (435)	13206-13640 (435)	13138-13572 (435)	TTT/TAA	TTG/TAA	TTG/TAG	
*nad4L*	13702-13941 (240)	13656-13895 (240)	13588-13827 (240)	ATT/TAA	GTT/TAA	ATT/TAA	
tRNA-Trp (W)	13919-13976 (58)	13873-13930 (58)	13805-13862 (58)				TCA

### Gene rearrangement in the mitochondrial genomes of *Ascaridia* species

**Figure 2:**
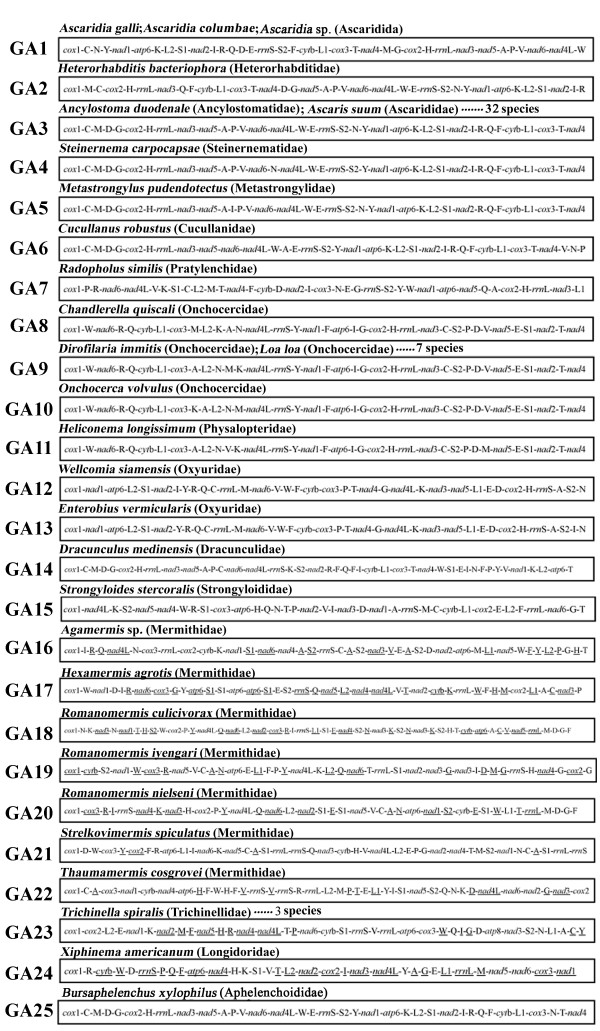
**Mitochondrial gene arrangement in three *Ascaridia *species (GA1) compared with those in other nematodes (GA2-GA25).** The circular mt genomes were linearized at the 5^′^ end of *cox1* gene for illustration purpose. Non-coding regions were not shown.

**Figure 3:**
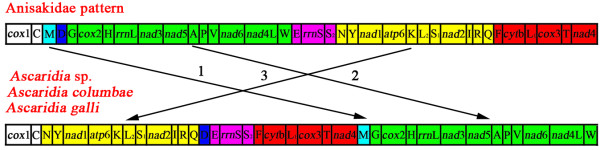
**Rearrangement of mitochondrial genes in three *Ascaridia *species (pattern GA1) relative to the most common pattern of mt gene arrangement observed in nematodes (GA3).**

*A. galli*, *A. columbae* and *Ascaridia* sp. (GHL-2012) have an identical gene arrangement in their mt genomes. The arrangement of mt genes in these three species, however, is substantially different from those of other nematodes (Figure 2). The mt genomes of the 62 species of nematodes that have been sequenced to date showed 25 types of gene arrangements (GA1–GA25, Figure 2), of which GA3 is the most common type and has been found in 32 species of nematodes. In comparison to the GA3 type, at least three rearrangement events occurred in the *Ascaridia* species: *trnM* was translocated, and a block of 11 genes (*trnN*, *trnY*, *nad1*, *atp6*, *trnK*, *trnL*_*2*_, *trnS*_*1*_, *nad2*, *trnI*, *trnR* and *trnQ*) swapped position with another block of 12 genes (*trnG*, *cox2*, *trnH*, *rrnL*, *nad3*, *nad5*, *trnA*, *trnP*, *trnV*, *nad6*, *nad4L* and *trnW*) (Figure 3).

### Phylogeny of nematodes inferred from mitochondrial genome sequences

#### Monophylies of the order Rhabditida and the subclass Dorylaimia

**Figure 4:**
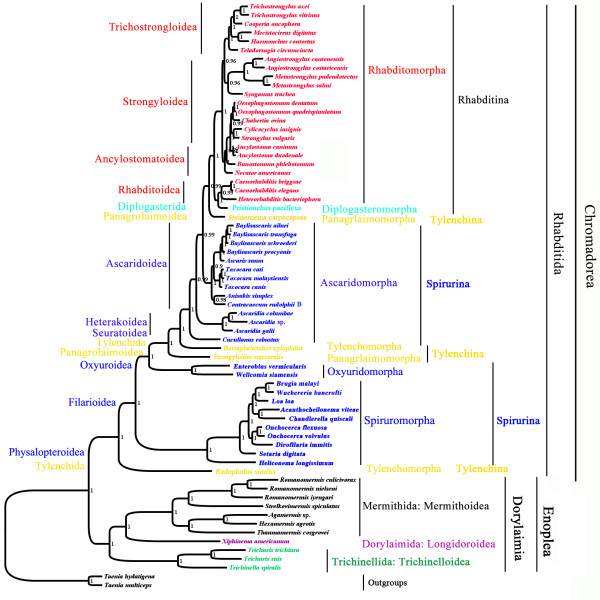
**Phylogenetic relationships among 65 species of nematodes inferred from Bayesian analysis of deduced amino acid sequences of 12 mitochondrial proteins.***Taenia multiceps* and *T. hydatigena* (GenBank accession numbers FJ495086 and FJ518620) were used as the outgroup. Posterior probability (PP) values were indicated at nodes.

**Figure 5:**
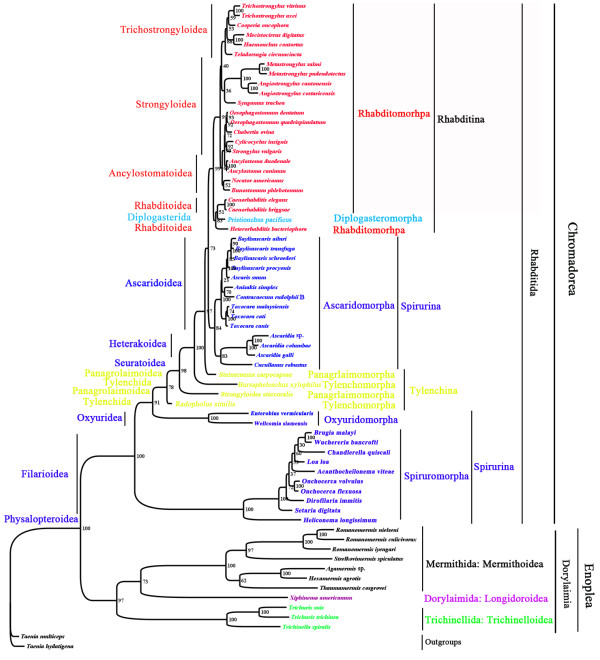
**Phylogenetic relationships among 65 species of nematodes inferred from maximum likelihood (ML) of deduced amino acid sequences of 12 mitochondrial proteins.***Taenia multiceps* and *T. hydatigena* (GenBank accession numbers FJ495086 and FJ518620) were used as the outgroup. Bootstrapping frequency (BF) values were indicated at nodes.

**Figure 6:**
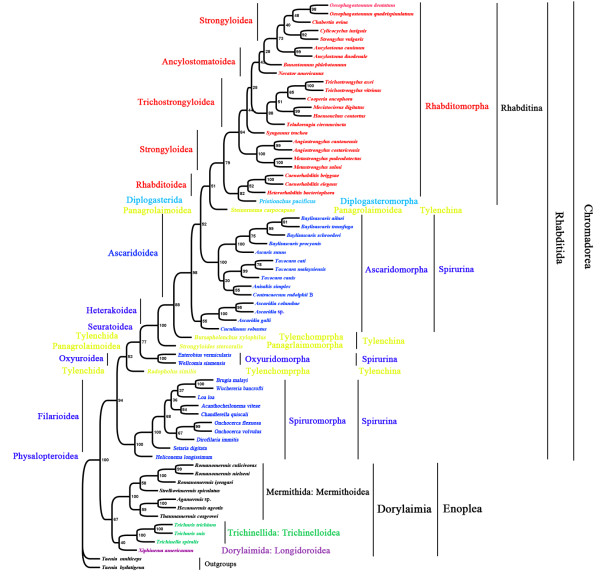
**Phylogenetic relationships among 65 species of nematodes inferred from maximum parsimony (MP) of deduced amino acid sequences of 12 mitochondrial proteins.***Taenia multiceps* and *T. hydatigena* (GenBank accession numbers FJ495086 and FJ518620) were used as the outgroup. Bootstrapping frequency (BF) values were indicated at nodes.

Of the 65 species of nematodes included in the phylogenetic analyses in this study, 54 species were from the order Rhabditida of the class Chromadorea, and 11 species were from the subclass Dorylaimia of the class Enoplea. Both Rhabditida and Dorylaimia were monophyletic in all of the trees inferred with Bayesian, ML and MP methods from concatenated amino acid sequences deduced from the sequences of the 12 mt protein-coding genes. The monophyly of the order Rhabditida was strongly supported with a posterior probability (PP) of 1 in Bayesian analysis (Figure 4), a bootstrapping frequency (BF) of 100% in ML analysis (Figure 5), and a BF of 94% in MP analysis (Figure 6). The monophyly of the subclass Dorylaimia was strongly supported in Bayesian and ML analyses (PP = 1, Figure 4; BF = 97, Figure 5), and was moderately supported in MP analysis (BF = 67%, Figure 6).

#### Phylogenetic relationships within the order Rhabditida

The 54 species of nematodes in the order Rhabditida included in this study were from three suborders: Rhabditina (24 species), Spirurina (26 species), and Tylenchina (4 species). The monophyly of the suborder Rhabditina was strongly supported in Bayesian and ML analyses (PP = 1, Figure 4; BF = 99%, Figure 5), and was moderately supported in MP analysis (BF = 79%, Figure 6). The Spirurina and the Tylenchina, however, were not monophyletic in all of the three phylogenetic analyses in this study.

Of the 24 species from the suborder Rhabditina, 23 species were from the infraorder Rhabditomorpha and one species from the infraorder Diplogasteromorpha. Species of these two infraorders were most closely related in all of the phylogenetic analyses in this study. The Rhabditomorpha, however, was paraphyletic with respect to the Diplogasteromorpha. Three species from the superfamily Rhabitoidea of the Rhabditomorpha were more closely related to *Pristionchus pacificus* (Diplogasteromorpha) than they were to the other 20 species from the Rhabditomorpha. The close relationship between the species of the superfamily Rhabitoidea and *P. pacificus* was strongly supported in Bayesian analysis (PP = 1, Figure 4), and was moderately supported in ML and MP analyses (BF = 83%, Figure 5; BF = 82%, Figure 6). In addition to the Rhabitoidea, three other superfamilies of the infraorder Rhabditomorpha were also represented in our analyses: Ancylostomatoidea (4 species), Strongyloidea (10 species), and Trichostrongyloidea (6 species). The Trichostrongyloidea was monophyletic with strong support in all of the three phylogenetic analyses in this study (PP = 1, Figure 4; BF = 88%, Figure 5; BF = 88%, Figure 6). The Rhabitoidea was monophyletic with strong support in Bayesian analysis (PP = 0.99) and weak support in MP analysis (BF = 52%, Figure 6), but was paraphyletic in ML analysis with weak support (BF = 51%, Figure 5). The Ancylostomatoidea and the Strongyloidea were paraphyletic in all of the three phylogenetic analyses in this study; there was strong support for the paraphylies of these two superfamilies in Bayesian and MP analyses (PP > 0.96, Figure 4; BF = 99%, Figure 5).

The 26 species of the suborder Spirurina included in this study were from three infraorders: Ascaridomorpha (14 species), Oxyuridomorpha (2 species) and Spiruromorpha (10 species). The Oxyuridomorpha and the Spiruromorpha were both monophyletic with strong support in all of the three phylogenetic analyses (PP = 1, Figure 4; BF = 100%, Figure 5; BF = 100%, Figure 6). The Ascaridomorpha, however, was monophyletic only in ML analysis with moderate support (BF = 84%, Figure 5), and was paraphyletic in Bayesian analysis with strong support (PP = 0.99, Figure 4) and MP analysis with weak support (BF = 52%, Figure 6). In both Bayesian and MP analyses, the 10 species of the superfamily Ascarodoidea of the infraorder Ascaridomorpha were more closely related to those of the Rhabditina and *Steinernema carpocapsae* (Tylenchina) than they were to the three *Ascaridia* species and *Cucullanus robustus*, which were also from the infraorder Ascaridomorpha. The three *Ascaridia* species, for which mt genomes were sequenced in this study, form a monoplyletic group with strong support in all of the three phylogenetic analyses (Figures 4, 5, 6). *Cucullanus robustus* was sister to Ascaridomorpha + Rhabditina + *Steinernema carpocapsae* in Bayesian analysis with strong support (PP = 1, Figure 4). *Cucullanus robustus*, however, was sister the *Ascaridia* species with moderate support in ML analysis (BF = 83%, Figure 5) and weak support in MP analysis (55%, Figure 6).

The four species of the suborder Tylenchina included in this study were from two infraorders: Panagrolaimorpha (2 species), and Tylenchomorpha (2 species). Both of these infraorders were paraphyletic in all of the three analyses (PP > 0.99, Figure 4; BF > 57%, Figure 5; BF > 51%, Figure 6).

#### Phylogenetic relationships within the subclass Dorylaimia

The 11 species of the subclass Dorylaimia included in this study were from three orders: Mermithida (7 species), Dorylaimida (1 species), and Trichinellida (3 species). The Mermitida and the Trichinellida were both monophyletic with strong support in all of the three analyses (PP = 1, Figure 4; BF = 100%, Figure 5; BF = 100%, Figure 6). Among the three orders, the Mermithida and the Dorylaimida were more closely related than either of them to the Trichocephalida, with strong support in Bayesian analysis (PP = 1, Figure 4) and moderate support in ML analysis (BF = 73%, Figure 5). The Dorylaimida was grouped with the Trichocephalida in MP analysis but the support to this grouping was weak (BF = 40%, Figure 6).

## Discussion

Phylogenetic relationships among nematodes have been revised in the past two decades using sequences of nuclear small subunit (SSU) rRNA gene [12-14]. The current working hypothesis of Nematoda phylogeny incorporated evidence from both morphology, ecology and nuclear SSU rRNA gene sequence analyses [14,15]. Recently, Kang *et al*. [23] tested the hypothesis of the Nematoda phylogeny with the mt genome sequences of 25 species. Kang *et al*. [23] showed strong support to the monophyly of the class Chromadorea, represented by 16 species from the order Rhabditida; this study, however, could not establish the monophyly of the class Enoplea, represented by nine species from the subclass Dorylaimia. Park *et al*. [24] and Sultana *et al*. [25] expanded the taxon sampling and inferred phylogenies with mt genome sequences of 36 and 41 species of nematodes respectively. Park *et al*. [24] showed strong support for the monophylies of both Chromadorea and Enoplea. Sultana *et al*. [25], however, could not establish the monophyly of the class Enoplea in most of their analyses. A major difference between the mt genome phylogenies and the current working hypothesis of the Nematode phylogeny is on the monophyly of the suborder Spirurina, which includes Clade III nematodes proposed in Blaxter *et al*. [12]. In the current working hypothesis, the suborder Spirurina is monophyletic, which includes the infraorders Ascaridomorpha, Spiruromorpha and Oxyuridomorpha, whereas the infraorder Rhabditomorpha is in the suborder Rhabditina [13,14]. Further, Ascaridomorpha is most closely related to Spiruromorpha, whereas Oxyuridomorpha is sister to the group that includes Ascaridomorpha + Spiruromorpha. In the phylogenies inferred from mt genome sequences, however, Ascaridomorpha is most closely related to Rhabditomorpha; in most analyses, Oxyuridomorpha is sister to Ascaridomorpha + Rhabditomorpha, whereas Spiruromorpha is sister to the group that contains Ascaridomorpha, Rhabditomorpha and Oxyuridomorpha. Kang *et al*. [23], Park *et al*. [24] and Sultana *et al*. [25] contained much less taxa compared to previous phylogenetic studies on nematodes with nuclear SSU rRNA gene sequences (e.g. >200 taxa in Meldal *et al*. [14]). The novel phylogenetic relationship inferred from mt genome sequences, thus, needs to be tested further with more taxa from a wide range of nematode lineages.

In the present study, we sequenced the complete mt genomes of three *Ascaridia* species from the family Ascaridiidae of the superfamily Heterakoidea, which was not represented in previous studies (Kang *et al*. [23]; Park *et al*. [24]; Sultana *et al*. [25]). Furthermore, we inferred the phylogenetic relationships among 65 nematode species, for which complete mt genomes have been sequenced to date. Our analyses support the division of nematodes into two classes, Chromadorea and Enoplea, represented by the order Rhabditida and the subclass Dorylaimia, respectively, in the present study. Both the Rhabditida and the Dorylaimia were monophyletic with strong support, regardless the methods of phylogenetic analysis used. Within the order Rhabditida, the suborder Rhabditina was monophyletic; the other two suborders, Spirurina and Tylenchina, however, were both paraphyletic in all of the three phylogenetic analyses in this study. Monophyly of the suborder Spirurina was well supported in nuclear SSU rRNA gene sequence analyses [12,14], but was rejected in mt genome sequence analyses in Kang *et al*. [23], Park *et al*. [24], Sultana *et al*. [25] and the present study. Phylogenetic analyses of mt genome sequences in all of these four studies support strongly a close relationship between Ascaridomorpha and Rhabditomorpha to the exclusion of Oxyuridomorpha and Spiruromorpha. Apparently, additional markers for phylogenetic inference are required to resolve the controversy on the Spirurina between mt genome sequence phylogeny and nuclear SSU rRNA phylogeny.

Gene arrangement in mt genomes provides a source of information for phylogenetic inference, independent from mt genome sequences [18]. It is noteworthy that 10 species of Ascaridomorpha and 20 species of Rhabditomorpha included in our phylogenetic analyses share a common pattern of gene arrangement, GA3, in their mt genomes (Figure 2). GA3 is also present in *Pristionchus pacificus*, the only species from the infraorder Diplogasteromorpha, which is closely related to the species from the superfamily Rhabitoidea of the Rhabditomorpha (Figures 4, 5, 6). Indeed, GA3 is the most common pattern of mt gene arrangement observed in nematodes to date; nevertheless, this pattern is present only in species of the Ascaridomorpha, the Rhabditomorpha and the Diplogasteromorpha. The possibility that GA3 is ancestral to the phylum Nematoda can be rejected with confidence thanks to the discovery of mt gene arrangement pattern GA23 in three *Trichinellida* species [29,30]. GA23 shares obvious similarity with that of many arthropods and would resemble the ancestral pattern of mt gene arrangement of the phylum Nematoda more than any other patterns found in the nematodes. There is no evidence either that GA3 is ancestral to the class Chromadorea. Given the strong support to the close relationship between Ascaridomorpha and Rhabditomorpha + Diplogasteromorpha indicated by mt genome sequence analyses in the present study, the most parsimonious explanation is that GA3 is a shared derived character (i.e. synapomorphy) of the clade Ascaridomorpha + Rhabditomorpha + Diplogasteromorpha. The other six patterns, GA1, GA2, GA4, GA5, GA6 and GA25, observed in either Ascaridomorpha and Rhabditomorpha or in species closely related to these two infraorders, can be derived from GA3 by only a few rearrangement events (Figure 2), e.g. GA1, observed in the three *Ascaridia* species, can be converted from GA3 by three rearrangement events (see above).

Monophyly of the suborder Tylenchina could not be established previously in nuclear SSU RNA gene sequence analyses [14]. No species from this suborder were included in two previous analyses of mt genome sequences [23,24]. The present study included four species from the Tylenchina and indicated the paraphyly of this suborder. The positions of the four species of the Tylenchina change depending on the methods of phylogenetic inference used in this study. For instance, *Steinernema carpocapsae* is sister to the suborder Rhabditina in Bayesian analysis (Figure 4) and MP analysis (Figure 6); it is, however, sister to Ascaridomorpha + Rhabditomorpha + Diplogasteromorpha in ML analysis (Figure 5). Further test with more species from this suborder apparently is needed in order to clarify the phylogenetic position of the suborder Tylenchina. Indeed, a very recent study by Sultana *et al*. [25] included six species from the Tylenchina and showed the paraphyly of this suborder with strong support. The paraphyly of the infraorder Rhabditomorpha with respect to the Diplogasteromorpha will also need further test with expanded taxon sampling as only one species, *Pristionchus pacificus*, from the Diplogasteromorpha was included in the present study. No species from the subclass Enoplia, which has two orders and eight suborders [13], was included in our analyses. For the subclass Chromadoria, all of the species included in this study were from the order Rhabditida whereas the other five orders, Plectida, Araeolaimida, Monhysterida, Desmodorida, and Chromadorida, were not represented. Expanding taxon sampling from these lineages of nematodes is clearly the next step for phylogenetic studies of nematodes with mt genome sequences.

## Conclusions

We sequenced the complete mt genomes of three *Ascaridia* species (family Ascaridiidae) that infest chickens, pigeons and parrots, respectively. These species have an identical arrangement of mt genes to each other but differ substantially from other nematodes. Phylogenetic analyses of the mt genome sequences of the *Ascaridia* species, in combination with 62 other nematode species, support the monophylies of the subclass Dorylaimia; the orders Rhabditida, Trichinellida and Mermithida; the suborder Rhabditina; and the infraorders Spiruromorpha and Oxyuridomorpha. Analyses of mt genome sequences, however, reject the monophylies of the suborders Spirurina and Tylenchina, and the infraorders Rhabditomorpha, Panagrolaimomorpha and Tylenchomorpha. Monophyly of the infraorder Ascaridomorpha varies depending on the methods of phylogenetic analysis. The Ascaridomorpha was more closely related to the infraorders Rhabditomorpha and Diplogasteromorpha (suborder Rhabditina) than they were to the other two infraorders of the Spirurina: Oxyuridorpha and Spiruromorpha. The closer relationship among Ascaridomorpha, Rhabditomorpha and Diplogasteromorpha was also supported by a shared common pattern of mitochondrial gene arrangement. Analyses of mitochondrial genome sequences and gene arrangement in the current study and several previous studies have provided novel insights into the phylogenetic relationships among several major lineages of nematodes. Many lineages of nematodes, however, are underrepresented or not represented in these analyses. Expanding taxon sampling is necessary for future phylogenetic studies of nematodes with mt genome sequences.

## Methods

### Ethics statement

This study was approved by the Animal Ethics Committee of Lanzhou Veterinary Research Institute, Chinese Academy of Agricultural Sciences (Approval No. LVRIAEC2011-009). The animals, from which the nematodes were collected *post-mortem,* were handled in strict accordance with good animal practices required by the Animal Ethics Procedures and Guidelines of the People's Republic of China.

### Sample collection and DNA extraction

Adult *Ascaridia galli* were obtained from naturally infected chickens in Hunan province, China. Adult *A. columbae* and an undescribed *Ascaridia* sp. [hereafter *Ascaridia* sp. (GHL-2012)] were obtained from naturally infected pigeons and parrots in Guangdong province, China. These nematodes were washed in physiological saline, identified preliminarily to species based on host preference, morphological characters and predilection sites [31-33], fixed in 70% ethanol, and stored at −20°C.

Total genomic DNA was isolated from individual nematodes using sodium dodecyl sulphate/proteinase K treatment, followed by spin-column purification (Wizard® SV Genomic DNA Purification System, Promega). In order to independently verify the identity of the specimen, the region spanning ITS-1, 5.8S rRNA gene and ITS-2 was amplified from each individual nematode by PCR using previously reported primers [34] and sequenced directly.

### PCR amplification of the mitochondrial genomes of *Ascaridia* species

We amplified fragments (400–500 bp) of *cox1*, *nad1* and *nad4* genes by PCR using conserved primers (Table 2). The amplicons were sequenced from both directions using BigDye terminator v3.1 on ABI PRISM 3730 platform. We then designed specific primers from these fragments for long PCR amplification (Table 3). We amplified the entire mt genome of individual specimens of *A. galli*, *A. columbae* and *Ascaridia* sp. (GHL-2012) by long PCR in three or four overlapping fragments, respectively. The three overlapping long-PCR fragments for *A. galli* were between *cox1* and *nad1* (~2.5 kb), between *nad1* and *nad4* (~7.5 kb), and between *nad4* and *cox1* (~5.0 kb). The four overlapping long-PCR fragments for *A. columbae* and *Ascaridia* sp. (GHL-2012) were between *cox1* and *nad1* (~2.5 kb), between *nad1* and *nad*3 (~5 kb), between *nad*3 and *cox2* (~2.5 kb) and between *cox2* and *cox1* (~5.0 kb). Each long-PCR reaction (25 μl) contains 2.5 μL 2 mM MgCl_2_, 4.0 μL 0.2 mM each of dNTPs, 2.5 μL 2.5 μl 10× rTaq buffer, 0.25 μL 2.5 μM of each primer, 0.25 μL 1.25 U rTaq polymerase (Takara), and 2 μl of total genomic DNA. The PCR conditions were: 92°C for 2 min (initial denaturation), then 92°C for 10 sec (denaturation), 48–51°C for 30 sec (annealing) and 60°C for 10 min (extension) for 10 cycles, followed by 92°C for 10 sec, 48–51°C for 30 sec, and 60°C for 10 min for 20 cycles and a final extension at 60°C for 10 min. PCR amplicons were column-purified and then sequenced using a primer walking strategy [35].

**Table 2:** **Primers used to amplify short-PCR fragments from *Ascaridia galli*, *Ascaridia columbae *and *Ascaridia *sp**

**Name of primer**	**Sequence (5′ to 3′)**	**Reference**
**For *A. galli***		
**p *cox *1**		
AGCox1F	GAAGTTTGTATTTGACTGGTAAGAA	This study
AGCox1R	CAGTGAGACCACCAATAGTAAACAA	This study
**p *nad *1**		
JB11	AGATTCGTAAGGGGCCTAATA	[36]
JB12	ACCACTAACTAATTCACTTTC	[36]
**p *nad *4**		
AGNad4F	CTTATTATTTAATTTTTTATGCTGCT	This study
AGNad4R	AAGCGGCTAAAGCCTTAGCATCACT	This study
**For *A. columbae *and *Ascaridia *sp.**		
**p *cox *2**		
ACCox2F	TTTAAGTTTGTTGTATTATTATGGTTT	This study
ACCox2R	AAAATACACCAACCACAGGAAAACT	This study
**p *nad *1**		
JB11	AGATTCGTAAGGGGCCTAATA	[36]
JB12	ACCACTAACTAATTCACTTTC	[36]
**p *cox *3**		
ACCox3F	TGGTATTTTCTGGACTTTTTTTGAT	This study
ACCox3R	CCAAACTACATCTACAAAATGCCAA	This study

**Table 3:** **Primers used to amplify long-PCR fragments from *Ascaridia galli*, *Ascaridia columbae *and *Ascaridia *sp**

**Name of primer**	**Sequence (5′ to 3′)**
**For *A. galli***	
AGC1N1F	ATAGAAGTTTGTATTTGACTGGTAAGAAGGAGGT
AGC1N1R	CACAATACCAGTAACCAAAGTAGCATAAACAG
AGN1N4F	TAAGTTGTTGAAGAAGGAGCAGGAGAGT
AGN1N4R	CAAAAATGGAAAAGAACACAAAGCAGCA
AGN4C1F	TTTTTATGCTGCTTTGTGTTCTTTTCCA
AGN4C1R	CCAAATAAAGTTGCCAGCCACCTAAA
**For *A. columbae***	
ACC1N1F	AGTTGGACTGTTTATCCGCCTTTGA
ACC1N1R	ATTTCATAAGACACTCTCTGACCTC
ACN1C3F	GCCAGAGGTCAGAGAGTGTCTTATG
ACN1C3R	CTTGCTTCACTATACTCTATTGCCTGT
ACC3C2F	CAGGCAATAGAGTATAGTGAAGCAA
ACC3C2R	ATAGAAGGCACAGCCCAAGAATGAA
ACC2C1F	TAGTATGTGATGTTTGGGAATGCTT
ACC2C1R	CTTTTACACCAGTAGGCACAGCGAT
**For *Ascaridia *sp.**	
ACC1N1F	AGTTGGACTGTTTATCCGCCTTTGA
ACC1N1R	ATTTCATAAGACACTCTCTGACCTC
ASN1C3F	GGTCAGAGGGTTTCTTATGAGATTGCT
ASN1C3R	CAACCGAACAATCTTTATTACTCAACA
ASC3C2F	TGTTGAGTAATAAAGATTGTTCGGTTG
ASC3C2R	ACCAGGAATGTCACCATACTCATAACT
ASC2C1F	TGGAGGTTGATAATCGTTGTGTTTTGC
ASC2C1R	CACAACAACAAAGGCTGAAAAACCATC

### Sequence assembly and mitochondrial genome annotation

Sequence reads of *A. galli*, *A. columbae* and *Ascaridia* sp. (GHL-2012) were assembled with ContigExpress program of the Vector NTI software package version 6.0 (Invitrogen, Carlsbad, CA). The mt genome sequences of the *Ascaridia* species were aligned with those of other nematode species available in GenBank using Clustal X 1.83 [37] to infer gene boundaries. The open-reading frames and codon usage profiles of protein-coding genes were analysed by the Open Reading Frame Finder (http://www.ncbi.nlm.nih.gov/gorf/gorf.html) using the invertebrate mitochondrial genetic code, and subsequently compared with those of *Ascaris suum*[38] and *Contracaecum rudolphii* B [28]. Sequences of protein-coding genes were translated into amino acid sequences using the invertebrate mitochondrial genetic code in MEGA 5.0 [39]. Translation initiation and termination codons were identified by comparison with those of the nematodes reported previously [27,28]. The secondary structures of 22 tRNA genes were predicted using tRNAscan-SE [40] with manual adjustment [41]. Tandem repeats in the non-coding regions were found using Tandem Repeat Finder program (http://tandem.bu.edu/trf/trf.html) [42].

### Phylogenetic analyses

Considering the high degree of intraspecific variation in nucleotide sequences of mt genes of nematodes [43], we used the deduced amino acid sequences of mt proteins for phylogenetic analyses. Amino acid sequences were deduced from the sequences of the 12 mt protein-coding genes common for all of the 65 nematode species and two outgroup species included in this study. The deduced amino acid sequences of each of the 12 mt proteins of these species were aligned individually and then were concatenated into a single alignment. Ambiguous sites and regions in the alignment were excluded using Gblocks (http://molevol.cmima.csic.es/castresana/Gblocks_server.html) [44] with the default parameters. Two *Taenia* cestodes (*T. multiceps* and *T. hydatigena*, GenBank accession number FJ495086 and FJ518620, respectively) were used as the outgroup.

Phylogenetic analyses were conducted using three methods: Bayesian, maximum likelihood (ML), and maximum parsimony (MP). The MtArt model of amino acid evolution [45] was selected as the most suitable model of evolution by ProtTest 2.4 [46] based on the Akaike information criterion (AIC). Bayesian analysis was with MrBayes 3.1.1 [47]. As MtArt model is not implemented in the current version of MrBayes, an alternative model, MtREV, was used in Bayesian analysis. Four independent Markov chains were run for 1,000,000 metropolis-coupled MCMC generations, sampling a tree every 100 generations. The first 2,500 trees represented burn-in, and the remaining trees were used to calculate Bayesian posterior probabilities (PP). Maximum likelihood (ML) analysis was performed using PhyML 3.0 [48] with the MtArt model. 100 bootstrap replicates were run and bootstrapping frequencies (BF) were calculated. MP analysis was with PAUP* 4.0b10 [49]; indels were treated as missing characters. A total of 1,000 random addition searches using tree bisection-reconnection (TBR) branch swapping were performed for each MP analysis. Bootstrapping frequency (BF) was calculated from 1,000 bootstrap replicates with 10 random additions per replicate in PAUP. Phylograms were drawn using FigTree v.1.31 (http://tree.bio.ed.ac.uk/software/figtree/).

## Competing interests

The authors declare no competing interests.

## Authors’ contributions

XQZ, RS and GHL designed the research. GHL and JYL performed the research. DHZ contributed reagents/materials/analyses. GHL, RS and HL analyzed the data. RS, GHL and XQZ wrote the manuscript. All authors have read and approved the final manuscript.
